# A practice-led assessment of landscape restoration potential in a biodiversity hotspot

**DOI:** 10.1098/rstb.2021.0070

**Published:** 2023-01-02

**Authors:** Abigail R. Wills, Deo D. Shirima, Olivier Villemaire-Côté, Philip J. Platts, Sarah J. Knight, Robin Loveridge, Hamidu Seki, Catherine E. Waite, Pantaleo K. T. Munishi, Herman Lyatuu, Blanca Bernal, Marion Pfeifer, Andrew R. Marshall

**Affiliations:** ^1^ Department of Environment and Geography, University of York, York YO10 5NG, UK; ^2^ National Carbon Monitoring Centre, Sokoine University of Agriculture, Morogoro, Tanzania; ^3^ Centre for Forest Research, Department of Forest and Wood Sciences, Université Laval, Québec, QC Canada, G1V 0A6; ^4^ BeZero Carbon Ltd, Discovery House, Banner St, London EC1Y 8QE, UK; ^5^ Leverhulme Centre for Anthropocene Biodiversity, University of York, York YO10 5DD, UK; ^6^ The Biodiversity Consultancy, Cambridge CB2 1SJ, UK; ^7^ Tanzania Forest Conservation Group, Dar es Salaam, Tanzania; ^8^ GreenCollar US, Chicago, IL, USA; ^9^ School of Natural and Environmental Sciences, Newcastle University, Newcastle upon Tyne NE1 7RU, UK; ^10^ Forest Research Institute, University of the Sunshine Coast, QLD 4556, Australia; ^11^ Reforest Africa, PO Box 5, Mang'ula, Kilombero District, Tanzania; ^12^ Flamingo Land Ltd, Kirby Misperton, North Yorkshire YO17 6UX, UK

**Keywords:** above-ground biomass, assisted natural regeneration, biodiversity conservation, climate change mitigation, forest landscape restoration, tree-planting

## Abstract

Effective restoration planning tools are needed to mitigate global carbon and biodiversity crises. Published spatial assessments of restoration potential are often at large scales or coarse resolutions inappropriate for local action. Using a Tanzanian case study, we introduce a systematic approach to inform landscape restoration planning, estimating spatial variation in cost-effectiveness, based on restoration method, logistics, biomass modelling and uncertainty mapping. We found potential for biomass recovery across 77.7% of a 53 000 km^2^ region, but with some natural spatial discontinuity in moist forest biomass, that was previously assigned to human causes. Most areas with biomass deficit (80.5%) were restorable through passive or assisted natural regeneration. However, cumulative biomass gains from planting outweighed initially high implementation costs meaning that, where applicable, this method yielded greater long-term returns on investment. Accounting for ecological, funding and other uncertainty, the top 25% consistently cost-effective sites were within protected areas and/or moderately degraded moist forest and savanna. Agro-ecological mosaics had high biomass deficit but little cost-effective restoration potential. Socio-economic research will be needed to inform action towards environmental and human development goals in these areas. Our results highlight value in long-term landscape restoration investments and separate treatment of savannas and forests. Furthermore, they contradict previously asserted low restoration potential in East Africa, emphasizing the importance of our regional approach for identifying restoration opportunities across the tropics.

This article is part of the theme issue ‘Understanding forest landscape restoration: reinforcing scientific foundations for the UN Decade on Ecosystem Restoration’.

## 1. Introduction

Forest landscape restoration has multiple potential benefits including enhancing biodiversity, carbon storage and the wellbeing and food security of people living in degraded landscapes [1]. The global goal to restore 350 million hectares of degraded land by 2030 is a reflection of the role of restoration in achieving sustainable development while combatting climate change and biodiversity loss. Timely realization of these goals is an enormous challenge. However, with global biodiversity and the terrestrial carbon sink in decline and ultimately on trajectories to disappear completely [2,3], enhancing carbon storage, biodiversity and associated ecosystem services through restoration is more important and urgent now than ever before. Systematic approaches are required to guide locally appropriate landscape restoration decision-making to deliver transparent, equitable and time-sensitive solutions for people and planet.

Accurate information on the spatial distribution and severity of landscape degradation, and thus ecological restoration potential, is needed to inform planning. However, research to this end is biased towards global [4–6] to national [7,8] level assessments that include input data, models and output maps of spatial resolutions that are too coarse to inform practical on-ground implementation [9]. This is also reflected in restoration planning tools, such as restoration opportunities assessment methodology [10] and FAO SEPAL, which are effective at guiding high-level strategies and priority-setting, but which are limited in their utility for finer scale, e.g. district-level and land-use planning [10]. Furthermore, failure to assimilate locally appropriate social and ecological knowledge and goal-setting has resulted in recommendations, and in some cases actions, that displace biodiversity (e.g. by planting trees in biologically important tropical grasslands and savannas; [11,12]) and local people [13]. Fine-scale habitat maps along with current and historical knowledge of local people, land use, tenure, governance, ecology and habitat dynamics are needed to inform effective restoration planning on the ground [4,14].

Practitioners also need access to information on appropriate methods for restoring degraded landscapes. The efficacy of different restoration approaches, i.e. passive or assisted natural regeneration (ANR) versus active restoration through direct seeding or planting native vegetation, varies in relation to local ecological conditions (e.g. previous land use; [15]) and landscape characteristics (e.g. distance from nearby intact habitats; [16]). These factors affect the likelihood of natural ecological regeneration [17], the rate of biomass accumulation [7] and the structure, type and diversity of species likely to regenerate [15,18]. Information to support method selection appropriate to varying scales of degradation and associated ecosystem properties exist from experimental studies and practitioner knowledge [19]. However, there are scant examples demonstrating how to apply this knowledge to inform spatially explicit landscape restoration planning.

Ecosystem restoration often incurs heavy costs, including direct financial cost of implementation and opportunity costs to local people [20,21]. However, with few exceptions, studies that incorporate direct financial costs into planning tend to apply approximate figures indiscriminately across entire landscapes [20,22,23], consider only a subset of costs (e.g. labour and material costs; [24]) and/or account only for costs incurred in the early stages of intervention [25]. By contrast, it can take 10 to 80 years to restore species composition [26,27] and 15 to greater than 1000 years to restore biomass [28]. In terms of opportunity costs, land rental, market prices for local produce, and population density are commonly used as proxies [25,29], but this fails to incorporate the full range of costs that restoration imposes on local people and on projects aiming to offset these. Thus, more complete, location-specific assessments of costs are needed to support restoration prioritization and decision-making that maximizes sustained social and ecological outcomes per unit investment [30].

Here, we develop and apply a systematic approach to inform spatially explicit forest landscape restoration planning. Our approach prioritizes cost-effective ecosystem recovery for timely achievement of global and regional restoration targets, accounting for biomass accumulation (and thus carbon sequestration and storage) objectives in a strategic region in Tanzania. The approach can be applied to any landscape-scale restoration project, using spatial prioritization methods for more detailed planning than is possible with existing restoration decision support tools. It is based on direct financial implementation costs of the most appropriate methods for restoring native vegetation and associated biomass, biodiversity, ecological function and livelihood options under different scenarios and investment time frames. In achieving this, unlike previous studies, we account for direct implementation and community engagement costs, logistics, expected vegetation growth and estimated uncertainty resulting from incomplete ecological knowledge. The findings are intended to be useful for advancing the science of restoration planning and for inspiring donors, through development of metrics directly useful for attracting and prioritizing grant funding.

## 2. Methods

**Figure 1.**
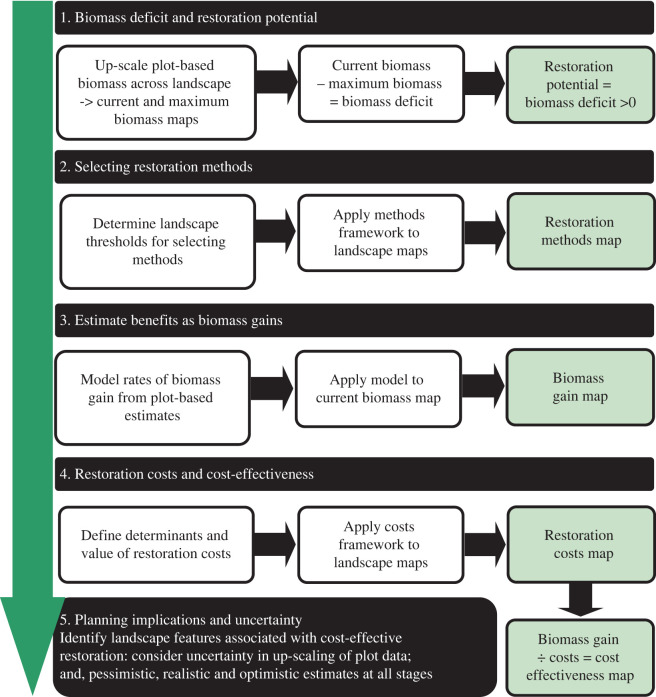
Stepwise approach for prioritizing areas for restoration based on assessments of direct financial costs and anticipated AGB gains upon implementing the most likely appropriate restoration methods, trialled in the Udzungwa-Kilombero Landscape, Tanzania. (Online version in colour.)

Our approach comprised four steps to determine the cost-effectiveness of ecological landscape restoration (figure 1). We incorporated pessimistic, realistic, and optimistic scenarios into all four stages and estimated above-ground biomass (AGB) gains, implementation costs and cost-effectiveness over two investment time frames: (i) 5 years, to represent a typical upper limit of donor investment; and (ii) expected time to full AGB recovery. AGB is one of the slowest attributes of tropical forest recovery [31], and we used this as a proxy for overall forest and biodiversity recovery on account of having good reference data. A combination of expert knowledge, pilot data and literature review were used to determine (i) environmental degradation thresholds for selecting methods, and (ii) comprehensive implementation costs.

### (a) Study region

We selected our study region for its biodiversity value, imminent threats, scientific knowledge-base and relevance for regional planning under an ongoing multi-stakeholder restoration effort [32]. The region comprised the Udzungwa and Mahenge Mountains and the Greater Kilombero Valley of Tanzania, hereafter ‘Udzungwa-Kilombero Landscape’ (53 000 km^2^; electronic supplementary material, figure S1; [33]). The Udzungwa-Kilombero Landscape forms part of the Eastern Afromontane biodiversity hotspot. It overlaps with a RAMSAR wetland, three national parks, two nature reserves, 31 national forest reserves, two forms of game reserve, an Important Primate Area, two Important Bird Areas and the Eastern Arc Mountains proposed World Heritage Site [34]. Landcover comprised a combination of human-dominated and primary, secondary and degraded native ecosystems, with high spatial variation in climate (rainfall 494–1938 mm yr^−1^; [35]), topography (elevation 108–2555 m; [36]), human disturbance [37] and habitat fragmentation resulting from population growth, historic settlements, logging and agriculture [38]. National and regional initiatives for supporting and incentivizing agricultural growth, e.g. ‘Kilimo Kwanza’ (Agriculture First) and the Southern Agricultural Growth Corridor Tanzania (SAGCOT), presented an additional threat to remaining habitat connectivity and attempts to restore it [39–41]. Lastly, the Udzungwa-Kilombero Landscape had been the focus of numerous scientific studies and charitable initiatives, many involving the authors, with an established network of vegetation monitoring plots (electronic supplementary material, figure S1) and considerable accumulated knowledge of the ecosystems and socio-political challenges.

### (b) Biomass deficit and restoration management

AGB (megagram per hectare) was estimated from 17 983 woody plant stems (trees, lianas, palms and stranglers) measured between 2007 and 2017 in 195 plots (electronic supplementary material, S2.2). For upscaling vegetation plot AGB estimates to map current AGB across the region, we used 2015 Google Earth Engine Landsat imagery comprising red, near-infrared and the two short-wave infrared (SWIR) spectral bands (30 m pixels; [42]). We used the raw reflectance data and derived two texture indices (the dissimilarity and average of the reflectance data, calculated using a moving window) for each of the four bands, following [43], giving a total of 12 potential predictors. A machine learning algorithm (random forest models) with 10-fold cross-validation and three repeats linked the satellite data to field-derived measurements of AGB. For this step, we excluded predictors that were highly inter-correlated (Pearson's *r* ≥ 0.6), keeping only those that correlated more highly with AGB. As such, the final model linking AGB to sensor data encompassed five variables: the SWIR 1 band, near-infrared band, and dissimilarity bands for red, near-infrared and SWIR 1. For the development and validation of the model, our dataset was split into training and test data at 4 : 1 ratio (156 : 39 plots). Maps of current AGB were converted to 25 m pixel rasters (UTM Zone 36S) which were then summed to form one hectare pixels.

Climate data were not used to predict current AGB across the landscape due to the unmeasureable effects of human influence on each pixel; however, they could be used to predict the maximum AGB obtainable for each pixel if managed towards a climax ecosystem. We related field AGB measures from a subset of closed-canopy plots to climatic predictor variables to map estimated maximum potential (hereafter ‘maximum’) AGB that could be achieved in the landscape based on climate constraints alone. We trialled the use of other biophysical (topographic: elevation, slope and aspect) variables alongside climate, but found that these were poor predictors compared to climate alone. Edaphic predictors were not included due to the lack of accurate data on soil characteristics in our study region. Climate data included 19 standard variables (electronic supplementary material, S2.2) for representing temperature, rainfall, moisture and seasonality, from Worldclim version 2 (30 arc second pixels; [35]), gridded to 3 arc second (approx. 90 m) resolution. Closed-canopy vegetation plot data (*n* = 59; mean size = 0.43 ha, range = 0.08–1.00 ha) were supplemented by randomly selected remotely sensed AGB estimates from our current AGB map, to balance the sampling of underrepresented ecosystems, in closed-canopy savanna spectrum areas (*n* = 33) and unlogged lowland forests (*n* = 10; less than 800 m.a.s.l.). To measure the climate–AGB relationship, we used random forest models equivalent to those used for spectral band modelling, with identical cross-validation and calibration to upscale maximum AGB. The final six predictor variables (Pearson's *r* ≥ 0.6) used in the upscaling (following [44]) were mean diurnal range, isothermality, driest quarter mean temperature, temperature seasonality, warmest quarter precipitation and maximum water deficit.

The expected loss of AGB since major human intervention in the landscape (AGB deficit) was then calculated by subtracting mean modelled current AGB from maximum AGB. All pixels where AGB deficit was greater than zero were considered to have potential to generate additional AGB, naturally or with silvicultural intervention, and thus to have ‘restoration potential’. Areas where AGB deficit was less than or equal to zero were assumed to have experienced gains or no change in AGB and thus were excluded from restoration planning.

### (c) Selecting methods and estimating biomass gain

For each hectare pixel with restoration potential, we assigned the most appropriate silvicultural approach for restoring AGB: passive regeneration, ANR or planting native vegetation (planting) (table 1). Key determinants of appropriate restoration methods included landcover class, elevation, degradation severity (using AGB deficit as a proxy; [57]) and Euclidean distance from nearest intact habitat, roads and/or disturbed habitat edges (using ArcGIS Pro version 2.7.1; [58]).

**Table 1.** Framework used to determine appropriate methods to restore above-ground biomass (AGB) deficit in the Udzungwa-Kilombero Landscape, Tanzania, adapted from proposed methods in relation to five degradation stages introduced by [19]. The framework includes realistic (with pessimistic and optimistic in parenthesis) estimates of landscape thresholds that can be applied to inform methodological decision-making for regional restoration planning, based on published research and pilot studies.

Restoration method	AGB deficit (%)	Elevation (m)	Distance (m)	Justification
Forest				
Passive regeneration	< 50 (< 40 to < 80)	Any	Any	In forests with low levels of degradation, vegetation was assumed to be sufficiently intact to self-regenerate in the absence of any silvicultural intervention. Our pilot observations indicate that this is the case in areas with < 50% (range = 40–80%) AGB deficit, beyond which secondary vegetation begins to hinder forest recovery. In such instances, cutting of vines, herbs and shrubs is effective at enabling tree recruitment, stem growth and net AGB gain [45,46]. In our region, *Lantana camara* invades forests (primarily at low densities such that it can be controlled by hand removal, [47]) along disturbed edges at low elevations located within 100–200 m of roads and/or agricultural areas
Vine cutting	≥ 50 (≥ 40 to ≥ 80)	< 1000	Any	
Herb/shrub cutting	≥ 50 (≥ 40 to ≥ 80)	≥ 1000	Any	
Lantana removal	≥ 50 (≥ 40 to NA)	< 1400	≤ 100 (≤ 200 to NA) disturbed	
Savanna/agro-ecological mosaic				
Passive regeneration	< 40 (< 30 to < 50)	Any	Any	Passive regeneration of degraded savanna can be hindered by dense grass and thicket, which hinders tree recruitment, growth and survival due to direct competition [48,49] and by increasing wildfire frequency and intensity [50,51]. To combat this, firebreaks and grass cutting were employed in areas with ≥ 40 ± 10% AGB deficit. Research shows that the land area affected by wildfires decreases dramatically where canopy cover exceeds 40 ± 10% [52]
Firebreak cutting	≥ 40 (≥ 30 to ≥ 50)	Any	Any	
Grass cutting	≥ 40 (≥ 30 to ≥ 50)	Any	Any	
Forest/savanna/agro-ecological mosaic				
Framework planting	≥ 65 and < 95 (≥ 50 and < 90; ≥ 80 and < 100)	Any	≥ 200 (≥ 100 to ≥ 300) intact	Based on AGB estimates for sites known by the authors to have significantly reduced ecosystem function in the study region, areas with ≥65 ± 15% AGB deficit were assumed to require planting to restore. Framework species planting [53] was planned in areas > 100–300 m from nearby intact forest (< 40–80% deficit) and savanna/agro-ecological mosaic (< 40–50% deficit), where seed dispersal was assumed to be restricted [16,17,54]. Severely degraded sites with ≥ 95 ± 5% AGB deficit were assumed to require soil improvement ([55] and [56]) followed by planting pioneer, nitrogen-fixing plants to be replaced with native trees over time [19]
Soil improvement and nurse planting	≥ 95 (≥ 90 to ≥ 100)	Any	Any	

Expected AGB gain per hectare from employing these methods was estimated using a regional dataset comprising cumulative modelled annual estimates of above-ground carbon (AGC, from zero to maximum) in naturally regenerating African forests, generated based on vegetation plot AGB measurement over time [59]. Firstly, modelled AGC values were converted to AGB, assuming 45.6 ± 2% AGC per unit of AGB [60]. AGB temporal change from this model was then used to estimate the proportion of maximum AGB expected after 20 years of restoration (mean = 0.51; range = 0.32–0.63), following standard guidelines for accounting landscape AGC change [61,62], and the average annual AGB increment per hectare: (i) for the first 20 years (mean = 7.28 Mg; range = 7.02–7.58), and (ii) from 21 years to full recovery (mean = 2.56 Mg; range = 1.54–4.11). We multiplied our maximum AGB map by the modelled proportion of maximum AGB at 20 years to estimate AGB stocks at that stage of recovery. These pixel values, along with modelled annual AGB increments up to and after 20 years, were compared to our current AGB map to estimate: (i) AGB gain after 5 years of restoration intervention, and (ii) the number of years needed to restore maximum AGB.

### (d) Restoration costs and cost-effectiveness

We estimated the financial cost of implementing restoration over our 5 year and complete recovery scenarios. We accounted for costs of (i) land procurement for restoration and tree nurseries; (ii) labour, equipment and transport for restoration, monitoring and management; (iii) engagement with communities close to human settlement, to understand local land use priorities and opportunities for complementary restoration; and (iv) project management and administration (electronic supplementary material, table S1). For restoration activities, we estimated cost per treatment per hectare and multiplied this by (i) number of treatments per year, and (ii) number of years of active management required to control factors inhibiting unassisted recovery of the remaining AGB deficit (electronic supplementary material, tables S2 and S3). We incorporated economies of scale into restoration cost estimates by making expert assumptions, based on practical experience from three regional initiatives, in regard to the likely land area that could feasibly be (i) restored at any one site; (ii) overseen by project staff; (iii) serviced by a single nursery (planting methods); and (iv) restored during each annual trip to the site (transport methods; electronic supplementary material, tables S1–S3). All costs were calculated in Tanzanian Shillings (TZS) and converted to US$ (2,326 TZS to 1 US$) accounting for inflation at 2.2% yr^−1^ [63]. We then calculated expected cost-effectiveness for per pixel:

2.1 $${\mathrm{AGB}}_{i}=\frac{\Delta {\mathrm{AGB}}_{i}}{i},$$

where, for time period *i*, AGB$ was the cost-effectiveness, i.e. expected gain in AGB per US$ (Mg US$^−1^), *Δ*AGB was the expected change in AGB and $ was the expected cost in US$.

We partially accounted for opportunity costs through calculating the financial costs of acquiring land for restoration and of socio-economic engagement. Detailed accounting of landscape-scale opportunity costs would require extensive spatial surveys of crop types, yields, sale prices and forecasted economic trajectories, which fell beyond the scope of this study. Furthermore, we considered opportunity costs to be best assessed through social surveys to understand and identify opportunities for restoration to complement local land-use goals and aspirations. Restoration may not be appropriate in locations with high opportunity costs [64].

### (e) Planning implications and uncertainty

Spatial variations in restoration potential, AGB gain, cost and cost-effectiveness were evaluated retrospectively in terms of technical implementation and landscape features of use to practitioners, namely: (i) restoration method, (ii) landcover class, and (iii) governance (protected areas, PAs, versus unprotected areas, NPAs). Means with standard deviations were used to summarise estimates of landscape AGB, which followed a broadly normal distribution, whereas costs and cost-effectiveness were described using medians and inter-quartile ranges. Cost-effectiveness of different methods, landcover and governance types was compared using Kruskal–Wallis tests with Dunn *post hoc* tests and Holm-adjusted *p*-values, *P*_adj_ [65]. Unless stated, results reflect findings from our realistic scenario, with results from pessimistic and optimistic scenarios in the electronic supplementary material (S3).

To account for uncertainty in our estimates, we report restoration potential before and after discounting areas with high ‘ecological uncertainty’ from climate modelling (defined using envelope uncertainty maps, EUMs, following [66]; electronic supplementary material, S2.4). We also identified the top 25% most cost-effective sites for restoration across all scenarios (pessimistic, realistic and optimistic) and investment time frames (5 years and to full recovery of AGB deficit) combined. We did this both overall within the landscape and specifically outside PAs, to identify locations with potential for community restoration schemes. All statistical and spatial analyses were conducted using R version 4.0.1 [67], besides the distance matrices and maps, which were produced in ArcGIS Pro version 2.7.1 [58]. The *caret* package was used for modelling [68] and the *raster* package for spatial up-scaling [69]. Statistical analyses were performed using the R *base* package. Both our R script (https://bit.ly/3KO5Hgz) and all input and output maps (https://bit.ly/3QkIrrI) produced during our stepwise method are available online.

## 3. Results

### (a) Biomass deficit and restoration management

Our map of estimated current AGB (electronic supplementary material, figure S2), up-scaled from spectral band models of vegetation plot data (*R*^2^ = 0.49; RMSE = 8.86; electronic supplementary material, figure S3), closely matched author familiarity with the region. For estimating maximum AGB, our climate model explained 75% of AGB variability across closed-canopy vegetation plots from primary forests and savanna woodlands (*R*^2^ = 0.75; RMSE = 73.98) with 54% of variation explained by mean temperature of the driest quarter and maximum water deficit (electronic supplementary material, figure S3). This yielded a maximum AGB map (electronic supplementary material, figure S2) with similar AGB in closed-canopy forests and savanna spectrum vegetation to our estimates of current AGB (electronic supplementary material, table S4). Based on our realistic scenario, we identified 77.7% (4.14 M ha) as having restoration potential, i.e. potential for passive or active biomass gain (table 2), mostly in savanna (49%; 2.03 M ha) and in the agro-ecological mosaic (45.5%), with a mean AGB deficit of 34.6 ± 0.26% across the landscape (table 2; electronic supplementary material, table S4). However, 10.9% (0.58 M ha) of the region had high ecological uncertainty (electronic supplementary material, figure S4), with the land area with restoration potential reducing to 68.5% (3.65 M ha) when discounting these areas. Still, there remained a high degree of additional uncertainty in our estimates, as illustrated by high variation between our pessimistic, realistic and optimistic current AGB, maximum AGB and AGB deficit maps (electronic supplementary material, figure S2).

**Table 2.** Restorable area and landscape restoration implementation costs (USD ha^−1^ yr^−1^), above-ground biomass (AGB) gains (Mg AGB ha^−1^) and cost-effectiveness (Mg AGB USD$100^−1^ ha^−1^ yr^−1^) over two investment time frames, 5 years (5Y) and to full recovery of maximum AGB (Full), in the Udzungwa-Kilombero Landscape, Tanzania. Data are presented overall and disaggregated by governance type (protected areas, PAs, versus unprotected areas, NPAs), land cover class and restoration method, including passive regeneration, assisted natural regeneration (ANR) and planting native vegetation. Median values are presented based on realistic assumptions in terms of AGB deficit between current and maximum AGB, most likely appropriate methods for restoring AGB, costs of employing these methods and AGB gained as a result, with additional measures of central tendency and variance provided in the electronic supplementary material, table S5.

Landscape variable	Area (%)	Area (M ha)	Cost ($US ha^−1^ yr^−1^)		AGB gain (Mg ha^−1^)		Cost-effectiveness	
			5Y	Full	5Y	Full	5Y	Full
Overall	77.7	4.14	6480	1188	12.8	77.6	0.092	8.78
Governance								
PA	29.0	1.20	6150	783	12.8	70.4	0.209	10.7
NPA	71.0	2.94	6510	1561	12.8	84.0	0.197	4.69
Land cover								
Moist forest	1.70	0.07	312	312	12.8	46.6	0.041	13.6
Savanna spectrum	49.0	2.03	5840	883	12.8	63.7	0.092	11.7
Floodplain	3.80	0.16	599	1010	12.8	38.1	0.091	9.91
Agro-ecological mosaic	45.5	1.88	44 500	2432	21.8	115	0.109	6.36
Silvicuture approach								
Passive regeneration	41.8	1.73	599	543	12.8	33.6	2.137	4.04
ANR	38.7	1.60	44 070	1656	17.6	99.4	0.070	5.92
Planting	19.5	0.81	52 850	2422	36.4	165	0.069	7.03

Due to having an AGB deficit of less than 50%, we estimated that 41.8% of the 4.14 M ha with restoration potential would regenerate passively (32.5% of the total land area; table 2; electronic supplementary material, figure S5). The remaining 58.2% (45.2% of the total land area) would require some form of silvicultural intervention to restore AGB, either through ANR (38.7%) or planting (19.5%). This increased to 71.6%, or 55.6% of the total land area, under our pessimistic scenario. Upon employing these methods (passive regeneration, ANR or planting), we estimated that it would take between 5.96 and 90.3 years (mean = 30.1 ± 17.1) to restore maximum AGB (based on modelled rates of AGB accumulation in naturally regenerating moist African forests; [59]), with 32.5 ± 23.5% of the AGB deficit (20.2 ± 9.75 Mg AGB ha^−1^) accumulated in the first 5 years and the remaining thereafter (electronic supplementary material, figure S5).

### (b) Restoration costs and cost-effectiveness

**Figure 2.**
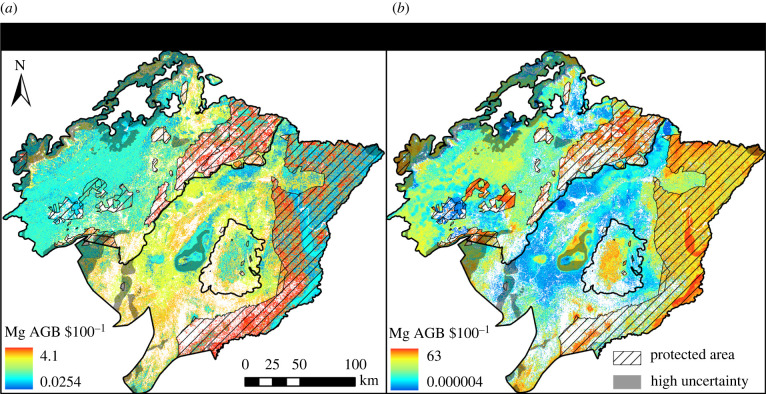
Cost-effectiveness in terms of above-ground biomass (AGB) gained per US$100 spent (Mg AGB $100^−1^) on restoration interventions in the Udzungwa-Kilombero Landscape, Tanzania, over (*a*) 5 years, and (*b*) through to full AGB recovery (30–90 years), based on realistic assumptions in terms of AGB degradation, appropriate methods, rate of AGB gain and direct financial costs. Locations of protected areas and areas of ecological uncertainty based on climate modelling, i.e. where one or more predictors were extrapolated beyond the 1/10th level [66], are indicated. (Online version in colour.)

We estimated that a median investment of $6480 ha^−1^ yr^−1^ would be required to fund restoration in the first 5 years, reducing to $1190 ha^−1^ yr^−1^ when considering time frames needed to restore maximum AGB (table 2). Returns on restoration investments increased over time; fully restored areas accumulated on average 8.78 Mg AGB ha^−1^ US$100^−1^, 95 times that gained in the first 5 years (figure 2; table 2).

**Figure 3.**
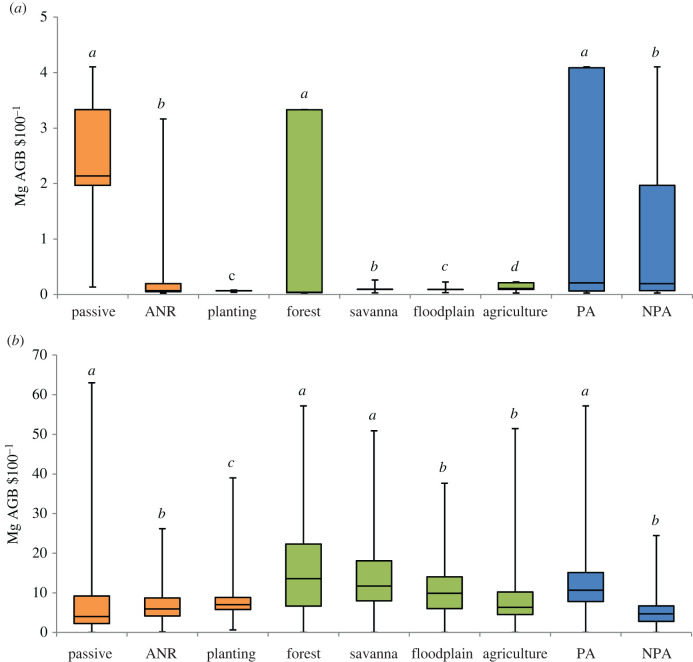
Cost-effectiveness (AGB Mg US$100^−1^ ha^−1^) of restoration interventions in the Udzungwa-Kilombero Landscape, Tanzania, disaggregated by (i) restoration method (passive regeneration, assisted natural regeneration, ANR, and planting); (ii) landcover class; and (iii) governance type (protected area, PA, versus unprotected area, NPA), over two investment time frames: (*a*) 5 years, and (*b*) to full recovery of maximum AGB. Methods, landcover classes and governance types with differing letters, *a*–*d*, have significantly different cost-effectiveness from Kruskal–Wallis tests, with Dunn *post hoc* tests as necessary (*P*_adj_ < 0.01). (Online version in colour.)

Passively regenerating sites were the least costly to manage, with little change in cost over time (table 2). Due to high-input costs required to raise, plant, manage and monitor seedlings (electronic supplementary material, table S3), planted areas were 20% more expensive than those restored through ANR and 87.2 times more expensive than passively regenerating sites in the first 5 years (table 2). However, planted sites accumulated AGB at a faster rate initially and more overall, on average becoming 19% and 74% more cost-effective in the long run than those restored through ANR and passive regeneration, respectively (table 2; figure 3; all differences significant, *P*_adj_ < 0.01).

Cost and cost-effectiveness also varied in respect to local land use and governance (table 2; figure 3). Governance structures significantly affected cost-effectiveness in both the 5 year and long-term scenarios (*P*_adj_ < 0.001). In the long term, restoration in PAs yielded 2.28 times more AGB per unit expenditure on average than that on unprotected land. Among landcover classes, forests were significantly more cost-effective for restoring maximum potential AGB than all other landcovers in the first 5 years (*P*_adj_ < 0.001). Over longer time frames, restoration of maximum potential AGB in forests remained 2.13 times more cost-effective than in the agro-ecological mosaic (*P*_adj_ < 0.001), but was not significantly more cost-effective than savanna (*P*_adj_ > 0.05).

### (c) Planning implications and uncertainty

**Figure 4.**
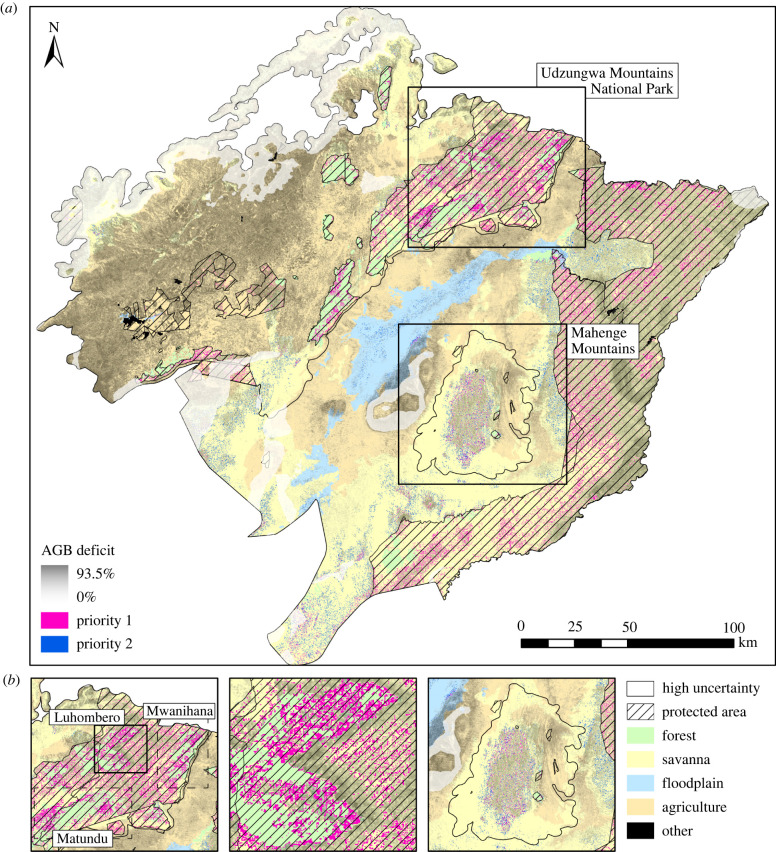
Location of primary and secondary priority areas for ecological restoration within the Udzungwa-Kilombero Landscape, Tanzania, defined as the top 25% for cost-effectiveness across all scenarios and investment time frames: (*a*) overall (pink, priority 1), and (*b*) outside protected areas (blue, priority 2). The distribution of landcover classes, protected areas and the proportional difference between current and maximum above-ground biomass (AGB deficit) are indicated alongside areas of ecological uncertainty based on climate modelling. (Online version in colour.)

We identified 3.34% of the landscape (4.30% of areas with restoration potential; 178 000 ha) that had both ecological certainty and top 25% cost-effectiveness across all modelled scenarios and investment time frames (figure 4; electronic supplementary material, table S6). Of these high priority areas, which constituted the most reliable and likely appropriate sites for prioritizing restoration to maximize returns on investment, 93.2% were within PAs, 77.9% comprised savanna, 18.8% comprised forest (savanna and forests accounted for 52.3% and 4.1% of total landcover, respectively) and 99.8% were restorable through passive regeneration or ANR. Restoration potential within PAs comprised 8.47% of the total land area under our optimistic scenario (451 000 ha), which could be used as a minimum target for future restoration management, due to combined ecological certainty and low opportunity costs. Savanna restoration, primarily through ANR, increased in priority over forests after discounting restoration potential in PAs, while restoration in the agro-ecological mosaic remained among the least cost-effective options (figure 4; electronic supplementary material, table S6).

## 4. Discussion

### (a) Spatial priority-setting

Our finding that the most cost-effective restoration sites coincided with protected yet moderately degraded forests and savanna has important implications for planning. Existing management structures and seed sources within these areas mean that costly additional forestry and community engagement staff and/or labour and resources for planting are not needed, thus reducing overall costs. Focusing restoration in PAs is also likely to result in fewer (additional) opportunity costs to local people, which can value at double that of implementation costs [25]. Furthermore, lessons-learned can be applied more broadly to inform best-practice restoration interventions across landscapes [70]. Thus, when the primary goal is to restore native woody vegetation within restricted budgets and time frames, we suggest that capitalizing on existing governance structures and natural assets is important for maximizing cost-effectiveness. Areas with these attributes could be immediately prioritized in order to meet time-sensitive global restoration targets.

A narrow focus on marginally degraded lands in PAs is likely to have limited additional benefits for improving connectivity for wildlife [71] and for direct benefits to local people. Low overlap in geographical restoration priorities for carbon storage, biodiversity and ecosystem services gains has also been reported elsewhere [8,72]. Furthermore, PAs accounted for a small proportion of the total restorable area in our region, meaning that there is a need to look to other areas to achieve restoration at necessary scales to combat biodiversity loss and climate change. At the landscape scale, as observed elsewhere [25], we showed that restoration of more degraded moist/wet forests (and also savanna woodlands in the longer term) was more cost-effective on average in comparison to riparian and multiple-use agro-ecological areas. Prioritizing restoration in moist forest environments, which are generally less favourable for cultivation than savanna [73], is likely to limit direct competition with local land use while still providing benefits (such as sustainable timber and firewood extraction; [74]). Furthermore, placing restoration focus on forests rather than drier woodlands also has the added benefit of prioritizing high-biodiversity ecosystems.

Prioritization of restoration based on anticipated biomass gains alone overlooks the value of restoring biodiversity and biomass in important drier ecosystems, which are generally less protected [75], increasingly vulnerable to extractions and clearance [73] and lacking in ecological restoration research [76]. This is supported by our finding that the majority of land with restoration potential coincided with savanna woodlands and/or the agro-ecological mosaic (which our maximum AGB estimates suggested to be converted savanna). However, as we have shown, restoring areas that improve connectivity in predominantly agricultural landscapes requires additional planning to minimize and/or offset opportunity costs (e.g. through sustainable use; [74]), which impacts cost-effectiveness. There is also potential for restoration in these areas to benefit human communities. For example, trees on farms can provide significant co-benefits to farmers by serving as additional sources of food and income [77], and by providing beneficial ecosystem services (e.g. soil microbes and pollination; [78]). While the recovery of moist forest in PAs will have positive benefits for ecosystem services—especially water, soil fertility and hence also food security—the direct benefits for livelihoods and wellbeing will be less predictable at a landscape scale, dependent upon a myriad of socio-economic drivers varying locally across the region. For this reason, our opinion is that human priorities for landscape restoration are best determined through direct, socio-economic surveys, rather than remote methods, and we view this as a major priority for future research. Thus, our emphasis on cost-effective restoration that maximizes AGB gains per dollar spent may therefore appeal more to donors aiming to meet global or regional restoration targets without compromising biodiversity or human livelihoods, and less so to those with more developmental goals. Prioritizing efforts in areas where these objectives overlap is important for meeting multiple climate, biodiversity and human development goals of landscape restoration [14,79].

Notwithstanding the extensive restoration potential in both forest and savanna, the agreement between our climate-modelled and current biomass in savanna suggests a high degree of natural isolation of moist forests. This is contrary to previous assertions of historical moist forest continuity in our study region which has been disrupted in the past 100 years [38] primarily due to human-induced fragmentation [80]. While some regional forest land has been converted to savanna, through clearance, grazing and fire [38], we assume that most of our forest–savanna mosaic is maintained by natural processes [81], including complex interactions between climate, fire, topography, soil [50,82,83] and mega-faunal activity [84,85]. Thus, our findings support that African savannas are an important global carbon store [86] and a naturally occurring landscape connectivity feature worth preserving rather than converting ‘back’ to forests, as has been proposed in our study region [87], elsewhere in Africa [11] and globally [88]. This underscores the importance of separate treatment of savannas and forests in landscape restoration plans [7] to conserve and promote landscape heterogeneity and diversity [89]. For improved input to spatial restoration modelling, planning and monitoring, permanent vegetation plots should thus aim to span all the vegetation types and chronosequences in a given region [90], including both old growth and secondary savanna [81].

Our modelled estimates of current AGB and forest and savanna distribution were comparable to the plot data, and reflected the authors' local knowledge of the region accumulated through on-ground observations over the past 23 years. This suggests that modelling approaches can be an effective tool for establishing biomass baselines and deficits for landscape restoration planning [89], when conducted at appropriate scales and supported by ground measurements for calibration. Thus, our approach is complimentary to coarser, larger-scale spatial projections of restoration potential, to better inform detailed planning. Finer-scale projections like ours, based on region-specific data, are also presumably more accurate, e.g. one recent global projection of restoration potential classified many East African rainforests as ‘non-restorable’, including all those in our study region [7], while another global projection used costs adjusted from a Brazilian study [91]. However, there is still considerable unpredictability even at our manageable regional scale, as illustrated by our uncertainty maps and high variation in restoration potential between scenarios, ranging from 8.5% to 55.6% of the study region. This emphasizes the importance of additional groundwork to map unmeasured ecological variability in larger-scale restoration mapping and to corroborate findings of regional restoration plans. Furthermore, while our AGB modelling projections appear intuitive, there is also potential for future experimentation. We did not account for variation in AGB accumulation rate as a result of restoration method [29], climate [92], topography [93], local site conditions (e.g. soil type, [49] and, former land use, [8]), or landscape features (e.g. presence of remnant vegetation, [94]), which can affect recruitment [94], growth strategies [95] and allometries [96]. Regional data were not available to enable these inclusions for all vegetation classes and modes of restoration, but are a research priority.

### (b) Cost-effective methods and investments

There is clear, under-used potential for passive regeneration and ANR in landscape restoration globally, which were applicable across more than 80% of the restorable area in our study landscape and incurred fewer costs than planting. Similarly, previous studies have shown ANR to be 0.38 [25] to 44 times [46] cheaper to implement than planting and applicable across vast areas [16], including a variety of vegetation types [97], and with comparable ecological outcomes to planting when prioritized appropriately [48]. This said, passive restoration is only feasible where ecological integrity has not been seriously disrupted, where planting becomes a crucial method for restoring degraded vegetation [19], e.g. approximately 19.5% of our restorable area. Planting also has potential to generate benefits for local communities, through job creation to raise seedlings and tend to restoration plantings, as well as to use nursery infrastructure for food and fibre production [19]. Thus, our findings support that a variety of methods are necessary to effectively restore landscapes, but that there is greater potential for restoration without planting than typically afforded in restoration planning [98] and implementation [7,48,99].

Our findings underscore the importance of measuring success in terms of long-term goals in order to maximize cost-effective outcomes for ecosystem restoration. Contrary to findings based on shorter-time frame studies [25,94], we found that sites restored through planting accumulated more AGB over time when compared to less severely degraded areas subjected to passive regeneration and ANR, and that these gains outweighed initially high implementation costs, thus increasing cost-effectiveness overall. Similarly, irrespective of the method employed, we demonstrate that overall cost-effectiveness increases with investment duration [29], by 95.1 times in our region, underscoring the need for long-term funding of restoration projects [64]. Similarly, investment size in terms of the contiguous land area restored can impact on cost-effectiveness, with potential 57% cost reductions as a result of increasing the size of restoration areas from one to 100 hectares [100]. This has important implications for communicating restoration priorities to donors and other investors in restoration, including local communities, since nature-based solutions need to be designed with long time frames in mind to restore species diversity, pre-disturbance AGB (and thus carbon stored) and for restored vegetation to play its key role in cooling the planet post peak global warming projected for the second half of the twentieth century [1]. Thus, we suggest that careful consideration of time frames, land area and economies of scale is needed for cost-effective restoration planning.

## 5. Conclusion

In response to urgent global calls for restoration that is sensitive to natural ecosystems, our emphasis on cost-effectiveness has highlighted key sites for restoration that will serve to maximize biomass gains in high-biodiversity areas. We show that restoration in moist forests and PAs, where existing governance structures and natural assets reduce implementation and opportunity costs, offers some of the best possibilities for cost-effective restoration in the immediate term. Passive regeneration and ANR are cheap to implement and are applicable on a large scale, supplemented by planting in more severely degraded areas. Longer-term investments exceeding 5 years are needed in both forest and savanna ecosystems, to successfully restore biomass to reference levels and this also improves overall cost-effectiveness, but will require a major shift in donor attitudes to grant funding, moving beyond short-term initial investments. Furthermore, a broader approach incorporating objectives for carbon, biodiversity, ecosystems and people will be needed for restoration to effectively contribute towards the multiple goals of ecosystem restoration, thus enhancing the financial, social and ecological feasibility of interventions. Nonetheless, our findings show that cost-effective locations for restoration of native ecosystems are widespread at a landscape scale, providing hope that this kind of approach can help to achieve international restoration targets through wise investments that maximize biomass gains at minimal cost. Key next steps will be to determine where restoration priorities for carbon storage overlap with priorities (and minimal opportunity costs) for biodiversity and human communities living in the landscape.

## Data Availability

Our dataset is available from the Dryad Digital Repository: https://doi.org/10.5061/dryad.nk98sf7wr [101]. Our R script is also available on GitHub (https://bit.ly/3KO5Hgz) and all input and output maps via figshare (https://bit.ly/3QkIrrI). Additional data are provided in the electronic supplementary material [102].
