# Correction to: ‘A practice-led assessment of landscape restoration potential in a biodiversity hotspot’ (2022) by Wills *et al.*

**DOI:** 10.1098/rstb.2022.0472

**Published:** 2023-02-13

**Authors:** Abigail R. Wills, Deo D. Shirima, Olivier Villemaire-Côté, Philip J. Platts, Sarah J. Knight, Robin Loveridge, Hamidu Seki, Catherine E. Waite, Pantaleo K. T. Munishi, Herman Lyatuu, Blanca Bernal, Marion Pfeifer, Andrew R. Marshall

*Phil. Trans. R. Soc. B* **378**, 20210070. (Published online 14 November 2022). (https://doi.org/10.1098/rstb.2021.0070)

In the original version of this article the authors' affiliations were listed incorrectly.

This has now been corrected on the publisher's website to the following:

Abigail R. Wills^1,†^, Deo D. Shirima^2,11^, Olivier Villemaire-Côté^3^, Philip J. Platts^1,4,5^, Sarah J. Knight^1^, Robin Loveridge^1,6^, Hamidu Seki^1^, Catherine E. Waite^10^, Pantaleo K. T. Munishi^2^, Herman Lyatuu^11^, Blanca Bernal^8^, Marion Pfeifer^9^ and Andrew R. Marshall^1,10,11,12,†^

^†^These authors contributed equally to this manuscript.

